# Sexual health needs and challenges among ageing women in Sub-Saharan Africa: a systematic review

**DOI:** 10.3389/fragi.2026.1803413

**Published:** 2026-08-21

**Authors:** Redson Mwandama, Yankho Henry Mdoka, Oudry Mulinde, Margubur Rahaman

**Affiliations:** 1 Department of Economics, School of Law, Economics and Government, University of Malawi, Zomba, Malawi; 2 Research Associate, Makoka and Associates Consulting Firm, Lilongwe, Malawi; 3 Research Consultant, Govind Ballabh Pant Social Science Institute, Prayagraj, India

**Keywords:** ageing women, menopause, reproductive health, sexual health needs, Sub-Saharan Africa, systematic review

## Abstract

**Background:**

The global ageing population is growing rapidly, with Sub-Saharan Africa undergoing a significant demographic transition. Although research on general ageing-related health outcomes in the region, such as frailty, multimorbidity, and socioeconomic deprivation, has expanded in recent years, the sexual health needs of ageing women remain comparatively understudied and inadequately addressed. This systematic review synthesizes evidence regarding the sexual health needs, challenges, and reproductive health concerns of ageing women in Sub-Saharan Africa.

**Methods:**

Scopus and PubMed databases were used to search, select, and review the publication records between 1 January 2000 and 26 May 2026. Sexual or reproductive health of women framed as ageing, older, elderly, menopausal, or postmenopausal in Sub-Saharan Africa studies were included. The PRISMA 2020 guidelines were followed for study selection and reporting. Data extraction and quality assessment were performed systematically.

**Results:**

A total of 358 records identified across Scopus and PubMed, 34 studies met the eligibility criteria and were included in the review. Six themes were identified: patterns and predictors of sexual activity in later life; menopausal symptom burden and sexual dysfunction; the intersection of HIV status and reproductive ageing; socio-cultural norms and the culture of silence; contraceptive needs and fertility ambivalence; and health-seeking behaviour and systemic barriers. Ageing women encounter multiple intersecting challenges, including age-related physiological changes, socio-cultural marginalisation, and inadequate responses from health systems.

**Conclusion:**

Sexual health needs of ageing women in Sub-Saharan Africa remain largely overlooked in research, policy, and healthcare delivery. There is an urgent need for age-sensitive sexual and reproductive health services, training for healthcare providers, and policy frameworks that acknowledge and address the unique needs of this population. Future research should prioritise empirical studies that highlight the lived experiences of ageing women and evaluate interventions aimed at enhancing their sexual health and wellbeing.

## 1 Background

Population ageing is becoming one of the most important demographic transitions worldwide. Although Sub-Saharan Africa remains the youngest region globally, the number of adults aged 65 years and older is projected to increase from 32 million in 2019 to more than 100 million by 2050 (Ani et al., 2023). This demographic shift is creating new demands on health and social care systems that have traditionally focused on infectious diseases and maternal and child health. Evidence from across Sub-Saharan Africa shows that older adults increasingly experience multimorbidity, frailty, disability, and socioeconomic deprivation, making healthy ageing an emerging public health priority (Kohler et al., 2024; Makofane et al., 2023; Metanmo et al., 2023; Mobolaji, 2024). Sexual health is an essential component of healthy ageing because it contributes to physical, psychological, and social wellbeing throughout life (Aboderin and Beard, 2015; Minichiello et al., 2012). However, the sexual health needs of older adults remain largely overlooked in research and healthcare, particularly in low- and middle-income countries.

Ageing women experience unique challenges related to menopause, sexual dysfunction, HIV risk, cervical cancer, and changing intimate relationships, all of which may affect their quality of life (Elias and Sherris, 2003; Negin and Cumming, 2010). Despite these challenges, sexual and reproductive health programmes in Sub-Saharan Africa continue to focus predominantly on younger women and maternal health. The situation is further complicated by social and cultural norms that discourage discussions about sexuality in later life. Many ageing women face stigma, gendered ageism, limited autonomy in reproductive decision-making, and barriers to accessing appropriate healthcare (Chepngeno-Langat and Hosegood, 2017; Mangipudi et al., 2020). Informal support and social networks that older women might otherwise rely on are themselves under strain in the region, further limiting the resources available to meet these needs (Banda et al., 2024; Hooley et al., 2023; Nutakor et al., 2023). In addition, healthcare providers often lack the training and resources needed to address the sexual health concerns of older women, contributing to unmet healthcare needs and limited utilisation of available services (Jonas et al., 2017).

These factors collectively make ageing women one of the most underserved populations within sexual and reproductive healthcare. Although research on ageing and women’s health has increased in recent years, most of that growth has concerned general physical and psychosocial ageing outcomes, such as frailty, adversity, and family wealth, rather than sexual health specifically (Jennings et al., 2024). The available evidence on the sexual health needs of ageing women in Sub-Saharan Africa remains fragmented across countries and health topics. To date, no comprehensive systematic review has synthesised this evidence to provide an overall understanding of their needs and challenges. Therefore, this review synthesises the existing literature on the sexual health needs, challenges, and experiences of ageing women in Sub-Saharan Africa to identify evidence gaps and inform future research, policy, and age-responsive healthcare interventions.

## 2 Methods

### 2.1 Protocol and registration

This review was conducted and is reported in accordance with the Preferred Reporting Items for Systematic Reviews and Meta-Analyses (PRISMA) 2020 statement (Page et al., 2021). A review protocol was not prospectively registered in PROSPERO or any other public registry. Although prospective registration is recommended to enhance transparency, it is not mandatory for conducting or publishing a systematic review. This is acknowledged as a limitation (Section 2.8). The review followed predefined eligibility criteria, search strategies, and methods throughout the review process.

### 2.2 Eligibility criteria

This review synthesises a single evidence base of primary empirical studies. Systematic reviews, meta-analyses, narrative reviews, and policy papers were excluded from the evidence base and are cited only, where relevant, as background literature in the Introduction and Discussion. Studies were eligible if they were (i) original quantitative (cross-sectional, cohort, randomised, or secondary analysis of survey data), qualitative, or mixed-methods research; (ii) focused on, or reported findings disaggregated for, women framed as ageing, older, elderly, menopausal, or postmenopausal, including studies drawing on a wider reproductive-age sampling frame where results are reported by an age band relevant to the ageing/perimenopausal transition; (iii) addressed a sexual or reproductive health outcome (sexual function, activity, or satisfaction; HIV/STI risk and prevention; intimate partner violence; family planning and contraception; cervical or HPV-related outcomes; menopause-related morbidity) as a primary or secondary focus; and (iv) were conducted in, or reported disaggregated data for, one or more Sub-Saharan African countries.

Studies were excluded if they were systematic reviews, meta-analyses, narrative reviews, conceptual or policy papers, conference abstracts, protocols without results, or dissertations; if they were conducted entirely outside Sub-Saharan Africa; if the primary outcome was a non-SRH health domain (e.g., bone density, general frailty, nutrition, or mortality unrelated to reproductive or sexual health) even where an older or HIV-positive cohort was described; or if ageing/older women were mentioned only in passing (e.g., as a comparator age band) without a disaggregated finding reported for that group.

### 2.3 Information sources and search strategy

Two databases were searched, Scopus and PubMed, for records published from 1 January 2000 to 26 May 2026; both searches were run on 26 May 2026. Search terms combined three concept blocks (population, phenomenon of interest, and Sub-Saharan African setting) with Boolean operators, tailored to each database’s syntax, with the population, outcome, and setting terms required to co-occur (AND) in the title/abstract. Scopus returned 72 records and the harmonised PubMed string returned 286 records, for a total of 358 records identified. Reference lists of included studies were also hand-searched for additional eligible records.

### 2.4 Study selection

**FIGURE 1:**
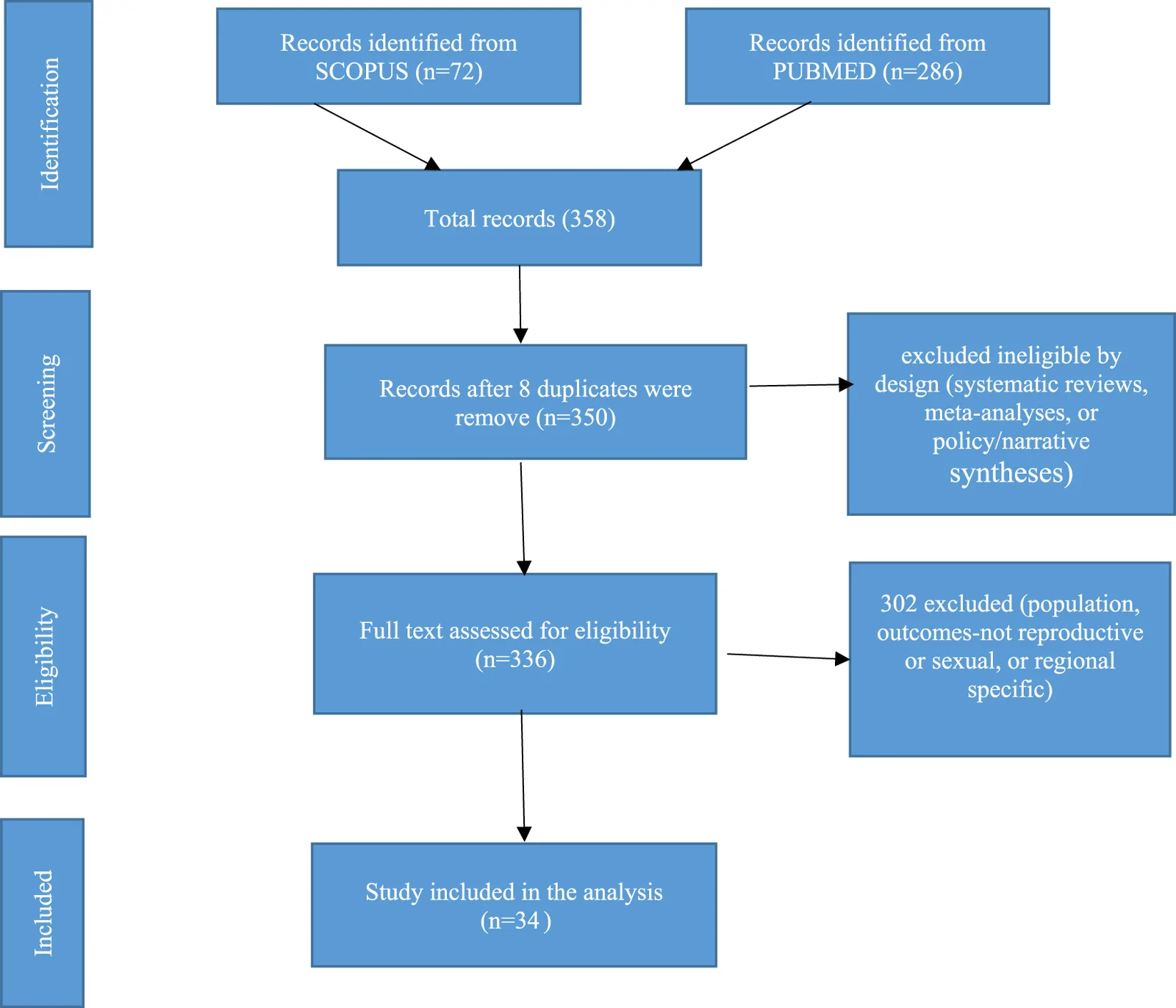
PRISMA 2020 flow diagram of study selection.

Because the harmonised search required population, outcome, and setting terms to co-occur, most retrieved records contained an ageing-women term, an SRH term, and a Sub-Saharan African country name somewhere in the text, including records where an ageing-women term appeared only in passing. Title/abstract screening therefore could not rely on keyword presence alone; each record was checked for whether ageing, older, or menopausal women were the actual focus of the reported finding, against the criteria in Section 2.2. Eight confirmed cross-database duplicates were identified by title-matching, leaving 350 unique records for title/abstract screening. Of these, 14 were excluded at this stage as ineligible by design (systematic reviews, meta-analyses, or policy/narrative syntheses), leaving 336 records for full-text retrieval and assessment. Following full-text assessment against the eligibility criteria in Section 2.2, 302 records were excluded, most commonly because the population was not centred on ageing or older women, the outcome was not sexual- or reproductive-health-specific, the study was not conducted in or disaggregated for Sub-Saharan Africa, or the record duplicated another included dataset. A total of 34 studies met all eligibility criteria and are included in this review (Table 1; Figure 1).

**TABLE 1:** Background characteristics of included studies (n = 34).

Author (Year)	Country	Study design	Setting	Sample size	Women’s age
Adedeji et al. (2024)	Nigeria	Descriptive cross-sectional	Community	420	40–65 years
Adedokun et al. (2009)	Nigeria	Descriptive survey	Urban community	254	60.3 years (mean)
Agaba et al. (2017)	Nigeria	Cross-sectional	Clinic	253	50 years (mean)
Agbeja et al. (2025)	Nigeria	Secondary analysis (MICS)	National household	11,248	35–49 years
Agunbiade and Gilbert (2020)	Nigeria	Qualitative	Urban community	125	≥60 years (mean)
Ahmed et al. (2026)	Nigeria	Secondary analysis (NDHS)	National household	4,884	15–49 years
Ande et al. (2011)	Nigeria	Cross-sectional	Community	533	57.4 years (mean)
Anyigor-Ogah et al. (2026)	Nigeria	Analytical cross-sectional	Hospital	160	65–98 years
Ayanore et al. (2020)	Ghana	Secondary analysis (WHO SAGE)	National	2,746	≥50 years
Chikwati et al. (2023)	Multi-country	Cross-sectional	Community	3,609	40–60 years
Cova et al. (2025)	Uganda	Cohort	Rural clinics	591	≥50 years
Debere et al. (2026)	35 SSA countries	Secondary DHS analysis	National	557,750	15–49 years
Dienye et al. (2013)	Nigeria	Hospital cross-sectional	Outpatient clinic	385	35–95 years
Drew et al. (2025)	Zimbabwe & South Africa	Qualitative	Urban communities	∼40	40–60 years
Dushimiyimana et al. (2025)	Rwanda	Cohort	Health facilities	1,546	≥55 years
Goyette et al. (2017)	Kenya	Prospective cohort	HIV clinics	404	47 years (median)
Harling et al. (2014)	South Africa	Population cohort	Rural community	1,734	30–50+ years
Madanhire et al. (2023)	Zimbabwe	Cross-sectional	HIV clinics	378	40–60 years
Mekonnen and Balemual (2023)	Ethiopia	Secondary analysis (EDHS)	National	1,628	35–49 years
Nkosi and Tsawe (2025)	Southern Africa	DHS pooled analysis	National	47,669	15–49 years
Obalowu et al. (2022)	Nigeria	Cross-sectional	Family medicine clinic	357	45–60 years
Odongo et al. (2025)	Uganda	Cross-sectional	Community	218	45–65 years
Okwuraiwe et al. (2025)	Nigeria	Comparative cross-sectional	Research institute	434	35–65 years
Roy et al. (2024)	Kenya, Rwanda & Uganda	Qualitative	Community	18	54–85 years
De Santo et al. (2025)	Kenya	Analytical cross-sectional	Community	1,042	8–90 years
Seffah et al. (2008)	Ghana	Retrospective	Hospital	93	≥50 years
Madiba and Ngwenya (2017)	South Africa	Qualitative	Primary health facilities	FGD participants	40–60 years
Taylor et al. (2010)	South Africa	RCT subgroup analysis	Trial sites	∼612	35–65 years
Effah et al. (2025)	Ghana	Retrospective	Screening centres	1,319	W ≥ 60 years
Tesfaw et al. (2025)	Ethiopia	Retrospective cohort	Oncology centres	∼400	≥50 years
Adedeji et al. (2026)	Nigeria	Mixed methods	Community	420 + 20 interviews	40–65 years
Shifera et al. (2025)	Ethiopia	Community cross-sectional	Community	404	57.7 years
Philip-Ephraim et al. (2010)	Nigeria	Hospital cross-sectional	Outpatient clinic	488	50–80 years
Saad et al. (2025)	Tanzania	Analytical cross-sectional	Municipal hospital	200	≥35 years

### 2.5 Data extraction

A standardised extraction form was applied to all 34 included studies, capturing author, year, country, study design, setting, sample size, population and age definition, and the main sexual/reproductive health domain addressed (Table 1). Each reviewer extracted data from a subset of studies, and a second reviewer independently checked all extracted data; disagreements were resolved by discussion.

### 2.6 Methodological quality assessment

Methodological quality was assessed using tools matched to study design: The Newcastle–Ottawa Scale (NOS) for cross-sectional, cohort, secondary/pooled-survey-analysis, retrospective, and randomised-trial designs (n = 29); the CASP Qualitative Checklist for qualitative studies (n = 4); and the Mixed Methods Appraisal Tool (MMAT) for the single mixed-methods study (n = 1). Quality assessment was conducted independently by two reviewers, with disagreements resolved by consensus. Across the 34 included studies, 29 (83%) were rated high quality and 5 (17%) moderate quality on their respective tool; no study was rated low quality (Table 2). Quality ratings informed interpretation of the synthesis (Section 3) but were not used to exclude studies from the narrative synthesis.

**TABLE 2:** Methodological quality assessment of included studies (n = 34).

Study type	Appraisal tool	Assessment domains	Overall quality
Observational studies (n = 29)	Newcastle–Ottawa Scale (NOS)	Selection, comparability, outcome/exposure	High (n = 24); Moderate (n = 6)
Qualitative studies (n = 4)	CASP Qualitative Checklist	Research aims, methodology, recruitment, data collection, reflexivity, ethics, analysis, findings, value	High (n = 4)
Mixed-methods study (n = 1)	Mixed Methods Appraisal Tool (MMAT)	Screening, qualitative, quantitative, and integration criteria	High (n = 1)

### 2.7 Data synthesis

Given the heterogeneity of designs, populations, and outcome measures across the 34 included studies, meta-analysis was not undertaken; a narrative synthesis was performed instead, following a Synthesis Without Meta-analysis (SWiM) approach. Studies were grouped thematically by the sexual/reproductive health domain most frequently reported: menopause and menopausal symptoms; sexual health, function, and sexuality; HIV and ageing; family planning and contraception; healthy ageing and multimorbidity; cervical/breast cancer screening; intimate partner violence; and prevention of mother-to-child transmission (Table 3). Within each theme, findings were compared across study designs and settings, and methodological quality (Section 2.6) was taken into account when weighing the strength of the evidence supporting each theme. A formal GRADE or GRADE-CERQual assessment was not conducted, given the narrative, non-meta-analytic synthesis across heterogeneous study designs; conclusions are framed accordingly, drawing on the study-level quality ratings (Table 2) and risk-of-bias ratings (Table 4) to weigh the strength of evidence supporting each theme.

**TABLE 3:** Sexual and reproductive health domains addressed (n = 34).

SRH domain	Number of studies
Menopause and menopausal symptoms	6
Sexual health, sexual function and sexuality	6
HIV and ageing	10
Family planning and contraception	4
Healthy ageing and multimorbidity	2
Cervical/breast cancer screening	4
Intimate partner violence	1
Prevention of mother-to-child transmission (PMTCT)	1
Total	34

**TABLE 4:** Risk of bias summary by domain.

Bias domain	Assessment	Evidence from included studies
Selection bias	Low to moderate	Most studies used probability sampling or nationally representative datasets; some hospital-based studies had limited representativeness
Information bias	Low to moderate	Several studies relied on self-reported sexual health measures, introducing potential recall and reporting bias
Confounding	Moderate	Most analytical studies adjusted for sociodemographic factors, although residual confounding remained possible
Reporting bias	Low	Outcomes were generally reported consistently with study objectives and analytical approaches
Overall risk of bias	Low to moderate	The majority of included studies were judged to have a low overall risk of bias, while a smaller proportion of hospital-based and descriptive studies were considered to have a moderate risk due to sampling limitations and self-reported outcomes

### 2.8 Limitations

This review has several limitations. First, the review protocol was not prospectively registered in PROSPERO or another public registry. Although prospective registration is recommended to enhance transparency and minimise the risk of selective reporting, it is not mandatory for conducting or publishing a systematic review. Nevertheless, predefined eligibility criteria, search strategies, and review methods were established and applied consistently throughout the review process. Second, a minority of the included studies drew on broad reproductive-age sampling frames rather than ageing-specific cohorts; findings from these studies were included only where results were disaggregated for ageing or older women. Finally, the definition of “ageing” or “older” women varied across studies (ranging from 35 years and above to 65 years and above, or using non-numeric descriptors), limiting direct comparability across the included studies and the overall synthesis.

### 2.9 Risk of bias summary by domain

Beyond the study-level quality ratings in Table 2, risk of bias was also appraised across four domains common to the included designs: selection, information, confounding, and reporting bias. Table 4 summarises the overall assessment for each domain together with the supporting evidence from the included studies.

Table 5 presents, for each of the 34 included studies, the main variables measured, the statistical approach used, and the principal finding reported. This evidence base underpins the six themes described in Section 3.

**TABLE 5:** Summary of evidence extracted from included studies (n = 34).

Author (Year)	Main variables measured	Statistical analysis	Principal findings
Adedeji et al. (2024)	Sexual health, finance, communication	Logistic regression	Poor sexual health associated with widowhood and low income
Adedokun et al. (2009)	Sexual activity, urological morbidity	Logistic regression	Sexual activity associated with education
Agaba et al. (2017)	HIV status, menopausal symptoms	Descriptive/bivariate	HIV linked with earlier menopause
Agbeja et al. (2025)	Contraceptive use	Multinomial logistic regression	Modern contraceptive use below 12%
Agunbiade and Gilbert (2020)	Menopause perceptions	Thematic analysis	Menopause viewed as protection from pregnancy
Ahmed et al. (2026)	Birth intervals	Logistic regression	Older women had longer optimal birth spacing
Ande et al. (2011)	Menopausal symptoms	Descriptive analysis	Joint pain and hot flushes most common
Anyigor-Ogah et al. (2026)	Functional status	Regression analysis	Lack of spouse associated with disability
Ayanore et al. (2020)	Cancer screening uptake	Ordinal logistic regression	Screening strongly influenced by wealth
Chikwati et al. (2023)	Cardiometabolic risk	Multivariable regression	West African women had highest metabolic risk
Cova et al. (2025)	HIV, inflammatory markers	Linear regression	Higher inflammatory biomarkers among women living with HIV
Debere et al. (2026)	Contraceptive use	Multilevel regression	Literacy and wealth predicted contraceptive uptake
Dienye et al. (2013)	Menopausal symptoms	Descriptive analysis	Most women sought informal treatment
Drew et al. (2025)	Menopause experiences	Thematic analysis	Menopause associated with physical and social changes
Dushimiyimana et al. (2025)	Dyslipidaemia	Modified Poisson regression	Older age predicted dyslipidaemia
Goyette et al. (2017)	Condom use	GEE models	Condomless sex declined after menopause
Harling et al. (2014)	HIV risk	Cox regression	Age-disparate relationships increased HIV risk
Madanhire et al. (2023)	Menopause symptoms, HIV status	Multivariable regression	HIV-positive women experienced more severe symptoms
Mekonnen and Balemual (2023)	Spousal violence	Logistic regression	Lower education associated with violence
Nkosi and Tsawe (2025)	Fertility preferences	Logistic regression	Desire to limit births highest among women aged 45–49
Obalowu et al. (2022)	Sexual activity	Logistic regression	Marriage predicted continued sexual activity
Odongo et al. (2025)	Menopause symptoms	Two-sample tests	Joint pain and hot flushes predominated
Okwuraiwe et al. (2025)	HIV status and menopause	Comparative analysis	Similar menopausal symptoms regardless of HIV status
Roy et al. (2024)	Gendered ageing	Thematic analysis	Older women reported social invisibility
De Santo et al. (2025)	Renal function	Logistic regression	Increasing age associated with renal impairment
Seffah et al. (2008)	Sexual function after surgery	Descriptive statistics	Sexual comfort improved after surgery
Madiba and Ngwenya (2017)	Condom negotiation	Thematic analysis	Patriarchal norms limited safer-sex negotiation
Taylor et al. (2010)	HPV infection	Kaplan-Meier analysis	Cryotherapy reduced HPV acquisition
Effah et al. (2025)	HPV screening	Chi-square tests	HPV positivity supported screening beyond age 65
Tesfaw et al. (2025)	Cervical cancer survival	Cox regression	Older age predicted mortality
Adedeji et al. (2026)	Vaginal dryness, libido	Mixed-methods analysis	Women reported unmet sexual health information needs
Shifera et al. (2025)	Menopause knowledge	Logistic regression	Poor knowledge associated with lower education
Philip-Ephraim et al. (2010)	HIV awareness	Descriptive statistics	Cultural barriers limited condom use
Saad et al. (2025)	PMTCT behaviours	Logistic regression	Older maternal age associated with better PMTCT behaviours

## 3 Key themes

Six themes emerged from the narrative synthesis of the 34 included studies (Table 3; Table 5). Each theme is summarised below with the key statistics reported by the underlying studies.

### 3.1 Patterns and predictors of sexual activity in later life

Sexual activity remains a significant but declining component of wellbeing for ageing women across Sub-Saharan Africa. Studies report sexual activity prevalence ranging from as low as 9.2% up to 40% among postmenopausal women (Adedokun et al., 2009). A primary predictor of continued sexual engagement is marital status, with married or partnered women significantly more likely to remain sexually active than widows or women without stable partners (Adedokun et al., 2009; Obalowu et al., 2022). While some women report no substantial changes in sexual frequency, approximately 68% of urban Nigerian women report reduced sexual activity following menopause (Obalowu et al., 2022). Factors contributing to declining sexual activity include partner unavailability, diminished sexual desire, and competition from younger co-wives in polygynous households (Agunbiade and Gilbert, 2020). Nevertheless, more than 80% of sexually active postmenopausal women describe their sexual experiences as pleasurable, underscoring the continued importance of sexual health and intimacy in later life (Obalowu et al., 2022).

### 3.2 Menopausal symptom burden and sexual dysfunction

Ageing women in SSA experience a substantial burden of menopausal symptoms that adversely affect sexual health and quality of life. Vaginal dryness and dyspareunia (painful sexual intercourse) are among the most frequently reported symptoms and are major contributors to sexual dissatisfaction, emotional distress, and strained intimate relationships (Adedeji et al., 2024; 2026). Evidence from Ethiopia indicates that the pooled prevalence of female sexual dysfunction (FSD) is 53.8%, with older age emerging as the strongest predictor (Shifera et al., 2025). In several settings, nearly all postmenopausal women report at least one menopausal symptom, with hot flushes, joint pain, muscle discomfort, sleep disturbances, and fatigue being particularly common (Ande et al., 2011; Dienye et al., 2013; Odongo et al., 2025). High levels of psychological distress, including depressive symptoms, further compound sexual health challenges and negatively influence overall wellbeing (Adedeji et al., 2026; Okwuraiwe et al., 2025).

### 3.3 The intersection of HIV status and reproductive ageing

HIV infection significantly influences the reproductive ageing experience of women in SSA. Studies show that women living with HIV are more likely to experience earlier menopause and report more severe menopausal symptoms than HIV-negative women (Agaba et al., 2017; Madanhire et al., 2023). HIV-positive women frequently experience heightened levels of irritability, anxiety, exhaustion, and somatic complaints, all of which negatively affect health-related quality of life (Madanhire et al., 2023; Okwuraiwe et al., 2025). While some evidence suggests that postmenopausal status is not necessarily associated with increased unprotected sexual activity among high-risk populations due to sustained risk-reduction counselling (Goyette et al., 2017), ageing women living with HIV remain vulnerable to a range of chronic health conditions. Emerging evidence indicates increased bone loss, chronic inflammation, dyslipidaemia, renal impairment, and elevated inflammatory biomarkers among older people living with HIV, highlighting the need for integrated HIV and geriatric care services (Cova et al., 2025; De Santo et al., 2025; Dushimiyimana et al., 2025).

### 3.4 Socio-cultural norms and the culture of silence

Socio-cultural beliefs strongly shape the sexual experiences of ageing women across SSA. In many communities, menopause is regarded as a socially acceptable endpoint to sexual activity, creating expectations of sexual withdrawal in later life (Agunbiade and Gilbert, 2020; Drew et al., 2025). Among Yoruba communities in Nigeria, menopause is often used as a cultural justification for refusing sexual relations due to beliefs that continued intercourse may result in “folk pregnancies” or other reproductive complications (Agunbiade and Gilbert, 2020). Such beliefs contribute to a pervasive culture of silence in which women feel embarrassed or reluctant to discuss sexual concerns with healthcare providers (Adedeji et al., 2026; Drew et al., 2025). Additionally, gendered ageism contributes to the marginalisation of older women, who frequently report feeling invisible, disempowered, and excluded from discussions about sexuality and reproductive health (Roy et al., 2024). In rural and patriarchal settings, fear of abandonment, stigma, or violence further limits women’s ability to negotiate safer sexual practices, including condom use (Madiba and Ngwenya, 2017; Philip-Ephraim et al., 2010).

### 3.5 Contraceptive needs and fertility ambivalence

Women of advanced reproductive age continue to face important reproductive health needs despite declining fertility. Evidence suggests that many women remain uncertain about future childbearing intentions, reflecting substantial fertility ambivalence during later reproductive life (Nkosi and Tsawe, 2025). Despite elevated maternal and neonatal risks associated with pregnancies at older ages, modern contraceptive use remains relatively low among women aged 35–49 years in many SSA countries (Agbeja et al., 2025; Debere et al., 2026). Contraceptive uptake is consistently associated with higher levels of education, wealth, media exposure, and access to healthcare information, while rural women remain particularly underserved (Debere et al., 2026). Among women who are not in marital unions, increasing age has been associated with a greater likelihood of contraceptive use, potentially reflecting greater autonomy and reduced social stigma compared with younger unmarried women (Agbeja et al., 2025).

### 3.6 Health-seeking behaviour and systemic barriers

Knowledge gaps regarding menopause and sexual health remain widespread among ageing women in SSA. Studies have reported that a substantial proportion of postmenopausal women possess limited knowledge about menopausal symptoms and available management options (Dienye et al., 2013; Shifera et al., 2025). As a result, many women rely on advice from peers, family members, or traditional healers rather than seeking formal healthcare services (Drew et al., 2025). Health systems across the region often remain focused on younger reproductive-age populations, and healthcare providers may assume that older women are sexually inactive, resulting in missed opportunities for sexual health counselling, screening for female sexual dysfunction, and STI prevention (Roy et al., 2024). Women frequently express a need for reliable information on sexual wellbeing, menopause management, and age-appropriate reproductive health services that are rarely available within primary healthcare systems (Adedeji et al., 2026). Furthermore, evidence of persistent high-risk HPV infections among women older than 60 years raises concerns about current cervical cancer screening cessation policies and highlights the need for age-inclusive screening strategies (Effah et al., 2025; Taylor et al., 2010).

## 4 Discussion

This review synthesised evidence from 35 studies conducted across Sub-Saharan Africa on the sexual health needs and challenges of ageing women. Twenty-nine of the included studies (83%) were rated high quality on their respective appraisal tool, and the overall risk of bias across the evidence base was judged low to moderate (Table 2; Table 4), giving reasonable confidence in the six themes identified. Sexual activity did not stop at menopause for most women, but it changed shape. Marital status was the strongest predictor of continued sexual activity across several studies, and most women who remained sexually active described the experience as pleasurable (Adedokun et al., 2009; Obalowu et al., 2022). At the same time, menopause brought a substantial symptom burden, vaginal dryness, dyspareunia, hot flushes, and joint pain, that was closely tied to sexual dissatisfaction and psychological distress (Adedeji et al., 2026; Ande et al., 2011; Shifera et al., 2025). Taken together, these two themes suggest that sexual health in later life is shaped less by age itself than by the interaction between physiological change, relationship context, and access to symptom management.

HIV infection added a further layer of vulnerability. Women living with HIV experienced earlier menopause, more severe symptoms, and a higher burden of chronic conditions such as dyslipidaemia and renal impairment than their HIV-negative peers (Agaba et al., 2017; Cova et al., 2025; Dushimiyimana et al., 2025). This points to a widening gap between HIV treatment programmes, which have extended life expectancy for people living with HIV, and geriatric and sexual health services, which have not kept pace with an ageing HIV-positive population. Socio-cultural norms consistently constrained how, and whether, ageing women could act on their sexual and reproductive health needs. Menopause was treated in several settings as a socially sanctioned endpoint to sexual activity, and gendered ageism left older women feeling excluded from conversations about sexuality (Agunbiade and Gilbert, 2020; Roy et al., 2024). Fear of stigma or violence further limited women’s ability to negotiate condom use (Madiba and Ngwenya, 2017; Philip-Ephraim et al., 2010).

These findings help explain a pattern that recurs across the health-seeking theme: modern contraceptive use remained below 12% in one national sample despite continued reproductive risk (Agbeja et al., 2025), and many women turned to informal sources of care rather than formal health services (Dienye et al., 2013; Drew et al., 2025). A culture of silence around older women’s sexuality appears to discourage both women and providers from raising the topic, compounding the low awareness reported in several studies (Shifera et al., 2025). The health system evidence reinforces this picture. Providers frequently assumed that older women were no longer sexually active, resulting in missed opportunities for sexual dysfunction screening, contraceptive counselling, and STI prevention (Adedeji et al., 2026; Roy et al., 2024). Persistently high rates of high-risk HPV infection among women older than 60 also raise questions about the appropriateness of current cervical cancer screening cessation policies in this region (Effah et al., 2025; Taylor et al., 2010).

These findings support three practical implications. First, sexual and reproductive health services in Sub-Saharan Africa need to extend beyond their current focus on younger, reproductive-age women to include age-appropriate counselling, symptom management, and screening for ageing women. Second, healthcare providers require specific training to recognise and address the sexual health needs of older women rather than assuming these needs disappear with age. Third, HIV care programmes for ageing populations should integrate menopause management and chronic disease screening rather than treating these as separate concerns.

This review should be interpreted in light of several methodological limitations. These include the absence of prospective protocol registration, variation in the definition of ageing across studies, and the inclusion of some studies based on broader reproductive-age populations with age-disaggregated findings. These factors may have influenced comparability across studies and the generalisability of the findings. The evidence base is also geographically uneven, with Nigeria contributing a disproportionate share of included studies relative to other Sub-Saharan African countries, which limits how far these findings can be generalised across the region. Future research should prioritise studies that centre the lived experience of ageing women directly, particularly outside West Africa, and should evaluate interventions designed to improve their sexual health and wellbeing.

## 5 Conclusion

This systematic review highlights that the sexual health needs of ageing women in Sub-Saharan Africa remain under-recognised in research, policy, and healthcare. Ageing women face multiple interconnected challenges, including menopausal symptoms, sexual dysfunction, HIV-related concerns, socio-cultural stigma, and limited access to age-appropriate sexual and reproductive health services. Strengthening age-sensitive healthcare, improving provider capacity, and integrating sexual health into routine care are essential to addressing these unmet needs. Future research should focus on underrepresented settings and evaluate interventions that improve the sexual health and wellbeing of ageing women.

## Data Availability

The original contributions presented in the study are included in the article, further inquiries can be directed to the corresponding author.
